# Performance assessment of different STPs based on UASB followed by aerobic post treatment systems

**DOI:** 10.1186/2052-336X-12-43

**Published:** 2014-01-27

**Authors:** Abid Ali Khan, Rubia Zahid Gaur, Indu Mehrotra, Vasileios Diamantis, Beni Lew, Absar Ahmad Kazmi

**Affiliations:** 1 Department of Civil Engineering, IIT Roorkee Roorkee, India; 2 Royal HaskoningDHV, New Delhi, India; 3 School of Environment and Natural Resources, Doon University, Dehradun, India; 4 Department of Environmental Engineering, Democritus University of Thrace, Xanthi, Greece; 5 The Volcani Center, Institute of Agriculture Engineering, Bet Dagan 50250, Israel; 6 Department of Civil Engineering, Ariel University, Ariel 40700, Israel

**Keywords:** Sewage, Upflow anaerobic sludge blanket reactor (UASB), Post treatment systems, Sewage treatment plants, Odour

## Abstract

This paper present the experiences gained from the study of ten up flow anaerobic sludge blanket (UASB) based sewage treatment plants (STPs) of different cities of India. Presently 37 UASB based STPs were under operation and about 06 UASB based STPs are under construction and commissioning phase at different towns. The nature of sewage significantly varied at each STP. Two STP were receiving sewage with high sulfate and heavy metals due to the mixing of industrial waste. The treatment performance of all UASB reactors in terms of BOD, COD and TSS were observed between 55 to 70% respectively. The post treatment units down flow hanging sponge (DHS) and Aeration followed by activated sludge process (ASP) at two STPs were performing well and enable to achieve the required disposal standards. Results indicate the effluent quality in terms of BOD and SS were less than 30 and 50 mg/L and well below the discharging standards.

## Introduction

The UASB technology has been widely employed for the treatment of sewage in Brazil, Columbia and India [1-4] since late 80’s. Nowadays it has been gaining popularity in other countries like United Arab Emirates (UAE), Angola and Indonesia etc. The experience gained with the application of UASB technology in India is unique and diverse. The India is a leading country in terms of volume of sewage treated by UASB process where 37 UASB based STPs is already operating [5-8]. It has been claimed that 80% of total UASB reactors installed worldwide for sewage treatment are in India. The basic approach towards selection of technology for sewage treatment is low capital costs, low energy requirements, low operation and maintenance (O & M) costs and sustainability aspect.

The BOD and suspended solids (SS) removal efficiencies from UASB reactor may vary from 55 to 75%. However, the UASB effluent BOD is higher than 60 mg/L and possibly up to 120 mg/L, the effluent SS concentration vary from 50 to 150 mg/L; FC removal is less than 1 logarithmic order and practically negligible removal for N and P and generally even a small increase which are well above the discharge standard limits [5,8]. Therefore, the UASB reactors generally require effluent polishing in order to comply with the disposal standards.

The polishing ponds (PP) are widely used as post treatment units since its inception, however, the performance of these post treatment systems were observed poor. According to Sato et al. [5] the polishing ponds were found ineffective to lower down the concentration of parameters of interest to the desired level. The treatment performance of the PP in terms of BOD and COD removal was between 21 to 25%, however, about 40% removal of SS was observed. The low HRT of 1-2 days might be the cause of poor performance of the PPs which is not optimum for such low rate systems [8].

Presently other aerobic post treatment systems such as the activated sludge process (ASP) and its variant like extended aeration (EA) followed by secondary clarifier are commonly installed for UASB effluent polishing. Modern systems DHS [9], Aeration [4,10] are developed for the polishing of UASB effluent. The performance of these systems is in most cases were ranged 60 to 80%.

The UASB technology is considered sustainable for environmental protection and resource recovery. Although UASB reactors are popular in India for sewage treatment, this is not the case for other countries. In these cases, it is important for the engineer to obtain reliable data (operational and design) from original UASB reactors in order to establish the proper treatment scheme.

Therefore, the objectives of this study was (i) to monitor the performance of full scale UASB reactors (ii) investigate the performance of existing post treatment systems (iii) to get an overview about the UASB based STPs working in the field with actual sewage.

Hence, this paper presents performance of ten full scale UASB based STPs along with different post-treatment options located at different part of the country.

## Materials and methods

### Description of STPs and monitoring plan

**Figure 1:**
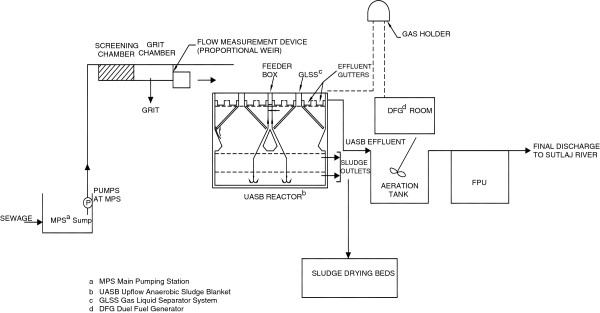
Schematic of STP process flow diagram (111 MLD Ludhiana).

All STPs monitored in this study adopted the sequence of units as screens-grit chambers-UASB reactors followed by one of the four post treatment: Final Polishing Unit (FPU) or polishing ponds (PP), and/ or other aerobic post treatment systems (Figure 1). The sludge drying beds, gas holder and dual fuel generators were common at all STPs beside one gas engine at a STP. The influent flow rates varied from 38 to 152 MLD (million litres per day).

The different STPs configuration and operational conditions were summarized in Table 1. The performance of STP’s 78 MLD-Agra, 40 MLD-Karnal, 43 MLD–Vadodara and 100 MLD- Surat were evaluated once based on grab sampling during the year 2008 and 2010. One STP (38 MLD- Saharanpur) was extensively monitored over a period of consecutive four years from 2007 to 2010 once or twice in each year. The performance of two STPs (27 and 34 MLD at Noida) was investigated twice in year 2010 and for remaining three STPs (48, 100 and 152 MLD at Ludhiana), the performance was monitored three times consecutively during the year 2010.

**Table 1:** Design parameters of STPs (UASB-Post treatment system)

**STPs**	**Capacity**	**UASB**				**Post treatment system**			
	**(MLD)**	**L × B × D/H (m)**	**No. of reactors**	**HRT (h)**	**Vol. (m**^**3**^**)**	**L × B × D/H (m)**	**No. of reactors**	**HRT**	**Vol. (m**^**3**^**)**
Saharanpur	38	24× 28× 6.10	4	9.4	15000	12700 m^2^ × 1.5 m (PP)*	2	1.0 d	38000
Agra	78	24× 40 × 5.25	6	9.3	30200	214 × 93 × 1.6 (PP)	1	1.2 d	97200
						130 × 160 × 1.6 (PP)	1	1.2 d	
						123 × 163 × 1.60 (PP)	1	1.2 d	
Karnal	40	32 × 24 × 4.8	4	8.5	14100	241 × 135 × 1.25 (PP)	1	1.0 d	40700
Vadodara	43	24× 22× 4.80	6	7.2	14680	52 × 26 × 4.0 (ASP)**	2	6 h	10816
Surat	100	20 × 20 × 7.44	20	8.5	39200	60 × 16 × 5.5 (ASP) **	4	3 h	21120
Noida	27	24 × 28 × 6.10	3	9.9	11200	110 × 120 × 1.6 (PP)	2	1.6 d	42000
Noida	34	24 × 24 × 6.25	4	9.6	13600	237.4 × 55.1 × 1.3 (PP)	20	1.0 d	38000
Ludhiana	111	32 × 30 × 5.1	9	9.0	44064	24 × 12 × 3.5 (Aeration)	1	10 min	1008
						602 × 270 × 1.5 (PP)	1	1.89 d	43810
Ludhiana	152	32 × 38 × 5.10	12	11	73836	602 × 270 × 1.5 (PP)	1	1.60 d	43810
Ludhiana	48	32 × 30 × 5.10	4	9.5	9.517002	602 × 270 × 1.5 (PP)	2	2.5 d	43810

*PP- Polishing Ponds, **ASP – Activated Sludge Process.

### Analytical procedure

Grab samples were collected from inlet chamber for sewage, UASB effluent at outlet pit, and final effluent from post treatment systems like polishing ponds (PP) or ASP etc. Samples were collected in one litre plastic bottles and kept in an air tight ice box and transported to the Environmental Engineering Laboratory, Department of Civil Engineering, IIT Roorkee, India.

Analysis of all physico-chemical parameters and heavy metals were made following the standard methods [11]. The samples for heavy metals analysis were collected into the 500 mL plastic bottles, which were acidified with 2 mL HNO_3_, in order to avoid the adsorption of heavy metals onto the wall of sample container.

## Results and discussion

### Sewage characteristics

The characteristics of sewage investigated at different STPs are presented in Additional file 1: Figure S1-S4*.* The data shows a significant variation in sewage strength. High BOD and COD concentrations were determined at five STPs (100 MLD-Surat, 27 & 34 MLD-Noida and 111 & 152 MLD-Ludhiana) however; only the COD concentration at 43 and 48 MLD STPs at Vadodara and Ludhiana was high (Additional file 1: Figure S2). TSS concentration was higher than 800 mg/L at two STPs -152 and 48 MLD, Ludhiana.

Sulfate concentration in sewage at 27 and 34 MLD STPs at Noida and 111, 152 and 48 MLD STPs at Ludhiana city was observed between 120 to 270 mg/L. The high sulfates concentration might be due to the disposal of industrial wastewaters into sewer. These values are higher than 20 to 50 mg/L generally found in sewage [12]. Mahmoud, 2002 [13] however, reported SO_4_^2-^ concentration as high as 900 mg/L in the sewage of Ramallah, Palestine.

The high BOD and COD concentration in sewage was attributed to the disposal of industrial waste since these STPs are located in highly industrialized areas.

### Performance of different UASB reactors

#### BOD, COD and TSS removals

The UASB effluent concentrations of BOD, COD and TSS along with their respective removal efficiencies are presented in Table 2. The highest removal of BOD, COD and TSS in UASB reactor was observed at five STPs viz. 38 MLD-Saharanpur, 40 MLD-Karnal, 43 MLD-Vadodara, 34 MLD-Noida and 111MLD-Ludhiana, and the final effluent concentrations ranged from 50-98, 139-277 and 89-128 mg/L for BOD, COD and TSS, respectively. The performance of the UASB reactors examined in this study was similar to well working UASB reactors reported in literature [4].

**Table 2:** Summary of treatment performance of UASB reactors

**STPs location**	**Capacity (MLD)**	**Mean effluent concentration (mg/L) and mean (%) removal efficiencies**		
		**BOD**	**COD**	**TSS**
Saharanpur	38	80 (60)	150 (55)	120 (60)
Agra	78	74 (48)	143 (43)	72 (41)
Karnal	40	68 (60)	163 (62)	89 (54)
Vadodara	43	57 (62)	139 (75)	114 (70)
Surat	100	135 (47)	402 (42)	142 (40)
Noida (Sector-50)	27	159 (53)	450 (41)	146 (59)
Noida (Sector-54)	34	50 (79)	277 (51)	128 (54)
Bhattian, Ludhiana	111	98 (66)	157 (59)	106 (64)
Balloke, Ludhiana	152	148 (59)	245 (55)	452 (49)
Jamalpur, Ludhiana	48	102 (45)	567 (29)	386 (51)

Values in parenthesis are percent removal efficiency.

The performance of three STPs at Agra, Surat and Ludhiana (i.e.78, 100 and 48 MLD) was not optimum and the removal of BOD, COD and TSS was 45-48%, 29-43% and 40-51% respectively. The reason for poor performance was improper O & M and lack of sludge wasting, grit removal and screening control. The performance of 27 and 152 MLD at Noida and Ludhiana reactors was observed relatively good with the BOD, COD and TSS removal efficiencies of 53-59; 41-55 and 49-59% respectively.

The high sulfates concentration in sewage did not result in process failure, however, high sulfides production was observed. According to Yamaguchi et al. 1999, the performance of the UASB reactors was barely affected at this sulfate concentration [14].

Comparatively the BOD, COD and TSS of sewage were observed high at these STPs. The removal of suspended solids in all UASB reactors was about 40-70%. The high TSS concentration in UASB reactors effluent was attributed to a limited amount of sludge disposal from the sludge bed. The removal of nitrogen and phosphorous was insignificant at all UASB reactors. The removal of pathogenic indicators TC and FC in UASB reactors was also observed low, in the order of 1 Log units.

### Odour problem at STPs

For many years, the odour nuisance has been of major concern at UASB based STPs, especially in cases of STPs surrounded by the densely populated cities. Although odour problems usually occur at different STPs, it is very intense at UASB STPs due to presence of H_2_S, major malodorous compound produced in UASB reactors. H_2_S gas becomes offensive at a threshold as low as 0.5 ppm [15]. The sulfides concentration at two STPs (34 and 111 MLD at Noida and Ludhiana) were monitored since the UASB effluent was high in sulfide concentration. The sulfides (H_2_S) concentration was measured by portable H_2_S meter (in air) at all units of these STPs especially surroundings of UASB reactor.

The concentrations of H_2_S at different locations are summarized in Additional file 1: Tables S1 and S2 at two STPs. High concentrations of H_2_S were observed near the aeration unit, gas holder and polishing ponds. This may be due the stripping of H_2_S in aeration and polishing ponds and leakage from the gas holder. At other locations of the STPs, very low concentration was measured.

### Methane generation rate

The batch experiments were conducted to evaluate the methane production rate from the sludge obtained from different UASB reactors. Glucose and sodium acetate were used as substrate to give a COD of 500 to 1000 mg. Experiments were conducted in Oxitop bottles. The results were summarized in Additional file 1: Table S3*. *The methanogenic activity test was carried out to determine the methane production potential of the anaerobic granular sludge taken from different UASB reactors from different port after thoroughly mixing. Results showed that the highest methane generation rate equal to 289 mL CH_4_/g VSS/ day, was observed at the 43 MLD Vadodara UASB reactor and 270 mL CH_4_/g VSS/ day at 111 MLD UASB sludge. The biogas generation rate of about 280-380 m^3^/h was measured at the gas flow meter at 111 MLD STP. Considering an average COD concentration of 500 mg/L the COD load is equal to 55000 Kg/d, or 2300 Kg/h. Biogas yield comes equal to 0.10-0.14 m^3^/kg COD feed or 0.17-0.23 m^3^/kg COD removed. This is relatively low but it is attributed to methane losses within the liquid effluent.

### Removal of heavy metals

The idea of investigating the heavy metals at three STPs of Ludhiana was evolve based on the earlier report published in state pollution control board of Punjab and moreover, Ludhiana is considered to be a highly industrialized city of India. The other STPs were not investigated for heavy metals since no evidence or such report occurred although trace amounts are always present in sewage which are considered as essential elements for the microbial growth and metabolism.

The heavy metals concentrations were determined in this study at 111, 152 and 48 MLD, STPs at Ludhiana from the influent, effluent and the digested dry sludge. Results show that the Pb and Zn concentration were high in the UASB effluent of 111 and 152 MLD STP however; the concentration of all heavy metals investigated was within the permissible limits for wastewater discharge to inland surface water bodies set by Central Pollution Control Board (CPCB) of India (see Additional file 1: Tables S4 and S5). The concentration of Cu, Co and Ni in the final effluent was higher than the EPA standards at the 111 MLD STP. The concentration of (Mn and Zn) and (Cr and Ni) were also higher compared to the EPA standards at 152 MLD and 48 MLD STP respectively.

The dry digested sludge of UASB reactors of 111, 152 and 48 MLD was also investigated for heavy metals. The concentration of Cu, Zn, Pb and Cd in dry sludge at 111 and 48 MLD plants were higher than the standard limits set by EPA (Additional file 1: Table S5)*.* Concluding, the treatment performance of the UASB reactors examined in this study was not adversely affected due to the presence of heavy metals.

#### Performance of existing post treatment systems of UASB effluent

Ten UASB reactors investigated at different STPs were utilizing four (4) different post treatment systems for effluent polishing. The following post treatment systems were installed within existing UASB STPs:

1. Down-flow hanging sponge (DHS) reactor.

2. Polishing ponds or Final polishing units (PP).

3. Aeration + Activated sludge process (ASP).

(a) Surface Aeration + Activated sludge process.

(a) Diffused Aeration + Activated sludge process.

4. Aeration + Polishing pond.

The DHS is a 1 MLD capacity demonstration plant installed at Karnal STP. The PP are widely used for UASB effluent upgrade and are currently in operation at five (5) STPs at Saharanpur (38 MLD), Agra (78 MLD), Karnal (40 MLD), Noida (27 MLD), Noida (34 MLD). Aeration + ASP as post treatment system is used for UASB effluent treatment at Surat and Vadodara. However, the mode of aeration is different at both STPs. Surface aerators are used at Vadodara STP while diffused aeration is used at Surat STP for aeration followed by ASP. Three STPs at Ludhiana are utilizing the aeration (surface aeration) + PP system for UASB effluent polishing. The operating conditions and design parameters of these post treatment systems are summarized in Table 1.

The performance of these post treatment systems with respect to the removal efficiencies of BOD, COD, and TSS along with final effluent values, are presented in Table 3. Results revealed that the attached growth DHS system was highly efficient for the removal of BOD, COD and TSS.

**Table 3:** Performance summary of different post treatment systems

**STP’s location**	**Post treatment system**	**Capacity (MLD)**	**Concentration mg/L (Mean removal efficiencies % in parenthesis)**						
			**BOD**	**COD**	**TSS**	**FC**	**NH**_**4**_**-N**	**NO**_**3**_**-N**	**PO**_**4**_**-P**
Karnal	DHS	43	13 (80)	21 (87)	10 (89)	2.3 + E02 (99.9)	11.4 (81)	4.5 (-)	1.05 (33)
Saharanpur	PP	38	36 (25-43)	51 (36-45)	40 (38-57)	2.3E + 03 (99)	28 (24)	2.77 (-)	3.33 (20)
Agra	PP	78	36 (52)	81 (43)	57 (21)	9.3E + 03 (99)	3.43 (76)	4.40 (-)	2.45 (31)
Karnal	PP	40	45 (34)	111(33)	60 (33)	9.3E + 03 (99)	55 (6.0)	4.93 (4.5)	9.0 (1.0)
Noida	PP	27	116 (27)	246 (46)	107 (27)	4.3E + 03 (99)	77 (15)	9.45 (-)	13.65 (-)
Noida	PP	34	46 (10)	164 (41)	85 (34)	9.3E + 03 (90)	71 (12)	5.15 (5)	14.4 (-)
Vadodara	Surface Aeration + ASP	43	13 (78)	35 (75)	21 (82)	4.3E + 04 (90)	14.67 (62)	2.7 (29)	3.97 (24)
Surat	Diffused Aeration + ASP	100	18.5 (86)	77 (81)	45 (65)	2.3E + 03 (90)	7.5 (82)	6.5 (-)	3.95 (2.5)
Ludhiana	Surface Aeration + PP	111	49 (31-56)	79 (50-60)	52 (27-37)	2.3E + 03 (99)	31 (21)	N.A	N.A.
Ludhiana	Surface Aeration + PP	152	93 (29-43)	136 (39-56)	239 (50-81)	2.3E + 04 (90)	39 (22)	N.A.	N.A.
Ludhiana	Surface Aeration + PP	48	46 (14-54)	242 (22-86)	185 (43-71)	2.3E + 04 (99)	26 (-)	N.A.	N.A.

(-) Insignificant removal and increase/ or decrease in values from initial values; N.A. - Not analyzed.

The 1 MLD capacity DHS reactor was installed at 40 MLD UASB based STP Karnal. The one MLD UASB effluent was used as an influent to DHS and the rest of the UASB effluent was treated by polishing pond. The DHS was operated at an HRT of 1.5 h. Sponge in cylindrical shape was used as media for microbial colonization.

### BOD, COD and TSS removals

**Figure 2:**
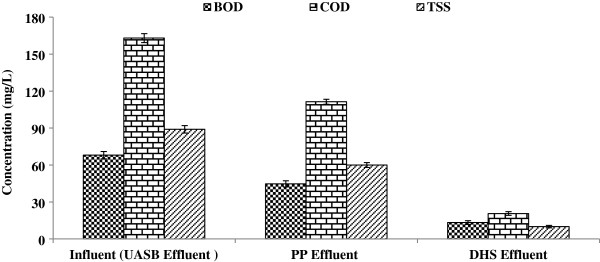
BOD, COD and TSS concentration of PP and DHS at Karnal STP.

High process efficiency for UASB effluent upgrade was achieved with the DHS system. The BOD, COD and TSS concentration values of the final effluent (UASB + DHS) were 13, 21 and 10 mg/L, respectively. The same UASB effluent was also treated by polishing ponds (PP) at same STP. Figure 2 revealed a clear distinction between the performances of these two post treatment systems. The treatment efficiencies were significantly lower in PP. In this case the final effluent was characterized by BOD, COD and TSS concentration of 45, 111 and 60 mg/L, respectively. The respective removal efficiencies are given in Table 3.

### Pathogens removal

Removal of pathogenic indicators during wastewater treatment is of major importance for both environmental and public health protection. National regulations for number of pathogens during treated wastewater disposal are strict, since the former directly affect public health [16]. In the present comparative study, total and fecal coliforms were used as indicator organisms for pathogens and were analyzed by the most probable number technique. Sewage coliforms concentration was in the order of 9.3 × 10^7^ MPN/100 mL. As it was expected the UASB reactor process does not significantly remove fecal coliform [17]. In this study, fecal coliform removal by the UASB reactor was approximately 1 Log. However, the removal of TC and FC in DHS were about 3–4 Log. The high removal of coliforms in DHS might be due adsorption in pores of the sponge media. The removal of TC and FC by the combined system was up to 5 Log. Final coliform counts in DHS effluent were 2.3 × 10^3^ and 2.3 × 10^2^ MPN/100 mL for total and fecal coliform respectively.

Coliform removal during UASB effluent treatment in PP was lower compared to the DHS system. The low detention time in PP causes the poor formation of the algae which reduced the removal of coliforms in PP. Total and fecal coliforms in the final UASB + PP effluent were 4.3 × 10^4^ and 9.3 × 10^3^ MPN/100 mL, respectively.

### Nitrogen removal

The removal of NH_4_-N in DHS was approximately 81% with final effluent concentrations of 11.4 mg/L. The respective NO_3_-N concentration was 4.5 mg/L. Nitrogen removal in the DHS system was higher compared to polishing ponds and aeration followed by polishing pond (Table 3). In UASB + DHS system, nitrification and denitrification was possibly responsible for low ammonia nitrogen and nitrates concentrations [18]. The ammonia was converted to nitrite and nitrate by nitrifiers which are then converted to gaseous nitrogen by denitrification in anoxic core of sponge material [19].

#### Final polishing ponds or units (PP)

Five (5) polishing ponds (PP) at different UASB based STPs were evaluated in this study. The PPs of the STPs monitored were at Saharanpur (38 MLD), Agra (78 MLD), Karnal (40 MLD), Noida (27 MLD) and Noida (34 MLD) respectively. One PP (38 MLD, STP) was studied extensively consecutively for four years in different seasons in order to evaluate the performance Table 4.

**Table 4:** Performance of Polishing Ponds (PPs) of 38 MLD STP Saharanpur

**Parameters**	**Feb 2007**			**Feb 2008**			**September 2009**			**January 2010**			**December 2010**		
	**UASB effluent**	**FPU effluent**	**% Avg. removal efficiency**	**UASB effluent**	**FPU effluent**	**% Avg. removal efficiency**	**UASB effluent**	**FPU effluent**	**% Avg. removal efficiency**	**UASB effluent**	**FPU effluent**	**% Avg. removal efficiency**	**UASB effluent**	**FPU effluent**	**% Avg. removal efficiency**
DO( mg/L)	0	4.75	-	0	1.58	-	0	3.15	-	0	3.15	-	0	1.70	-
pH	7.22	7.78	-	6.98	7.83	-	7.30	7.83	-	7.00	7.77	-	7.33	7.63	-
Alkalinity	359	377	-	355	352	-	346	376	-	343	375	-	289	269	-
BOD (mg/L)	72 ± 1.52	46 ± 2.82	36	68 ± 0.7	50 ± 0.57	25	89 ± 1	59 ± 4.16	33	80 ± 0.7	45 ± 1.52	43	51 ± 1.73	26 ± 2.88	48
COD (mg/L)	121 ± 1.73	75 ± 6.36	38	157 ± 1.41	100 ± 2	36	158 ± 2	99 ± 0.57	38	150 ± 0.7	85 ± 4.35	43	98 ± 11.68	54 ± 4.16	45
TSS (mg/L)	104 ± 1.15	49 ± 2.82	52	79 ± 0.7	48 ± 1.52	38	141 ± 1.52	67 ± 0.57	52	120 ± 0.7	53 ± 2.08	56	58 ± 2.51	39 ± 2.51	33
VSS (mg/L)	58	29	-	36	55	-	90	35	-	63	35	-	27	20	-
NH4-N (mg/L)	54 ± 1	41 ± 4.94	-	50 ± 1.27	53 ± 0.57	-	39 ± 0.57	51 ± 1.15	-	48 ± 0.7	43 ± 2.64	-	37 ± 1	27 ± 1.52	-
NO3-N (mg/L)	2.07 ± 0.05	3 ± 0	-	2.6 ± 0.14	3.03 ± 0.057	-	1.97 ± 0.057	2.93 ± 0.11	-	2.55 ± 0.07	3.2 ± 011	-	1.50 ± 0.2	2.63 ± 0.25	-
PO4-P (mg/L)	5.80 ± 0.1	4.75 ± 0.07	-	4.85 ± 0.07	5.70 ± 0.1	-	5.13 ± 0.2	4.60 ± 0.01	-	5.55 ± 0.07	4.80 ± 0.17	-	4.13 ± 0.05	3.27 ± 0.11	-
ORP (mV)	-	-		−157	99	-	−137	71	-	−167	75	-	41	133	-
Sulfates (mg/L)	14 ± 1.52	41 ± 4.94	-	15	24.67 ± 0.57	-	18 ± 0.57	0.43 ± 0.4	-	22 ± 0	26 ± 0.57	-	35 ± 3.05	37 ± 1.0	-
Sulfides (mg/L)	7.90 ± 0.1	0	-	8	4.67 ± 0.28	-	4.37 ± 0.32	25 ± 2	-	5.65 ± 0.07	1.77 ± 0.25	-	4.80 ± 0.55	3.50 ± 0.1	-

Two PPs at Noida (27 and 34 MLD, UASB based STPs) were investigated at different sampling points (see Additional file 1: Figures S5 and S6). An overview of different PP performance concerning the removal of BOD, COD, and TSS is presented in Table 3.

### BOD, COD and TSS removals

**Figure 3:**
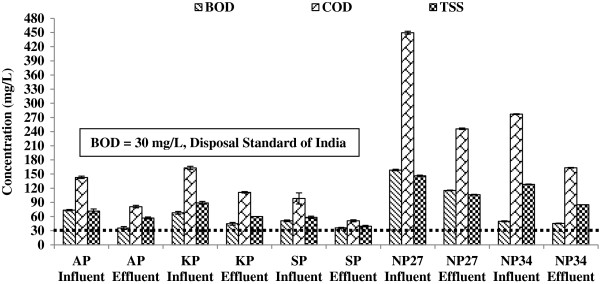
BOD, COD, TSS concentration in PP.

The removal of organic matter and suspended solids was similar in different polishing ponds. Especially, BOD, COD and TSS removal efficiency varied between 10-52, 33-46, and 21-57%, respectively (Figure 3). The final effluent was characterized by BOD: 36-116 mg/L; COD: 51-246 mg/L and SS = 40-107 mg/L. Results of this study revealed that the performance of the examined polishing pond does not achieve BOD and TSS disposal standards. This is attributed to the low hydraulic retention time (1 to 2 days). The removal of suspended solids was similar to the organic matter.

### Pathogens removal

TC and FC counts in the PP influent were in the order of 4.3 × 10^4^ to 9.3 × 10^5^ MPN/100 mL. The removal of TC and FC in PP was about 1–2 Log and the final effluent was characterized by 2.3 × 10^3^ and 9.3 × 10^3^ MPN/100 mL, respectively.

The mechanisms responsible for the removal of TC and FC in PPs include high pH, high DO values (frequently reaching super saturation) and UV penetration especially in shallow PPs. From the Table 3, it is evident that none of the examined PPs was lower than 1,000 MPN/100 mL, which is the limit for unrestricted irrigation according to the WHO standards.

Although the PP examined in this study revealed extremely low efficiency, properly designed natural treatment systems can achieve high purification degree for municipal wastewater [20].

### Nitrogen and phosphorous removal

Insignificant removal of ammonia (6-24%) was observed at different PPs except for the Agra STP. In this case, the removal of NH_4_-N reached 76%. The final ammonia nitrogen concentration is high (which is beneficial for agricultural reuse or problematic for disposal in sensitive water bodies). Phosphorus removal was also negligible. Anaerobic reactors in general do not remove P, while substantial P removal in PP is possible by precipitation at high pH values [21].

### Evaluation of PP at different points

Data concerning the performance of two polishing ponds (STPs 27 and 34 MLD, Noida) monitored at different sampling location are presented in Table 5. Nine (9) sampling points were selected to evaluate the mechanisms for the removal of BOD and TSS. The exact location of different sampling points in two different seasons i.e., autumn (20 October 2010) and winters (5^th^ January 2011) were shown in Additional file 1: Figures S5 and S6. The HRT of these ponds varied from 1 to 2 days. The performance of the ponds depends mainly on temperature, dissolved oxygen and pH. The removal of BOD, COD and SS was mainly due to physical processes (settling of solids or particulate BOD). The average HRT of these ponds was ~ 1 day. Accordingly, the removal of nutrients and pathogens was limited. In general, one day HRT is not considered sufficient for algal growth [20]. The percentage removal of BOD, COD and SS in WSP varied between 20-30%, 25-35% and 30-40% respectively. The final effluent was characterized by a BOD and SS concentration was between 40-50 mg/L.

**Table 5:** **Summary of data investigated at different locations of Two STPs FPU (20**^**th **^**October 2010)**

**FPU capacity**	**FPU - 27 MLD STP**							**FPU-34 MLD STP**							
**Sample/parameters**	**Inlet Channel to FPU 3**	**Inlet of FPU 4**	**FPU location 5**	**FPU location 6**	**FPU location 7**	**FPU location 8**	**Final Effluent**	**Inlet to FPU**	**FPU location 1**	**FPU location 2**	**FPU location 3**	**FPU location 4**	**FPU location 5**	**FPU location 6**	**Final effluent**
Temp. (^°^C)	29.7	29.9	28.8	31	33.9	28.9	28.4	28.3	28.4	29.2	29	28.7	28	28.8	29.3
pH	7.2	7.2	7.5	7.9	7.5	8.9	8.1	7.2	7.4	7.6	7.4	7.5	7.8	7.8	7.6
Alkalinity as CaCO_3_ (mg/L)	700	728	660	700	648	720	648	680	688	676	680	660	664	676	632
BOD (mg/L)	72	69	68	60	56	53	48	84	83	83	82	80	77	71	68
COD (mg/L)	208	207	205	165	145	132	118	261	253	242	236	232	225	196	192
TSS (mg/L)	178	144	132	120	111	101	91	102	94	88	76	70	66	63	60
VSS (mg/L)	101	101	56	50	43	40	36	56	47	41	40	38	34	33	32
NH_4_-N (mg/L)	49.3	49.5	49.7	31.2	20.7	20.0	24.0	90.5	92	87	84	82	80	75	71
NO_3_-N (mg/L)	3	2.8	2.8	2.9	3.3	3	4.6	2.5	3.2	3.3	3.1	2.89	2.9	3.9	3.9
PO_4_-P (mg/L)	4.2	4.2	4.4	4.0	4	3.6	3.6	7.6	7.9	7.6	6.6	6.8	5.8	6	5.7
ORP (mV)	−331.2	−340.0	−367.7	−142.5	−53.5	−42.4	−40.2	−344	−390	−400	−399	−389	−399.9	−394.5	−393
DO (mg/L)	0.96	0.85	0.42	6.56	9.48	20.43	17.28	0.6	0.13	0.16	0.22	0.12	0.16	0.13	0.14
Sulfides (mg/L)	24	32	32	0	0	0	0	64	52	26.4	28	28	24	24	22
Sulfates (mg/L)	47	49	53	59	58	57	58	136	155	163	190	204	200	178	175

The results of this study indicate that the polishing ponds used for UASB effluent upgrade were not designed properly and their performance was limited to a clarification process.

### Aeration + Activated sludge process

#### Diffused aeration + activated sludge process followed by secondary clarifier

A diffused aeration + activated sludge process was used for UASB effluent polishing at 100 MLD STP, Surat. Aeration was performed by fine pore diffusers installed at the bottom of the aeration tank. The DO concentration in the aeration tank was maintained at 0.8 - 1.0 mg/L. The size of the aeration tank was 65 × 20 × 3.5 m. Sewage temperature ranged between 26 to 33°C.

The final BOD and TSS concentrations were 18.5 and 45 mg/L, respectively, below the disposal standards. The removal of ammonia nitrogen was higher than 80% but FC removal was approximately 1 Log. The average mixed liquor suspended solids (MLSS) concentration was maintained at 2300 to 2500 mg/L. The SV30 of aerobic sludge was equal to 450 mL/L resulted in sludge volume index (SVI) of 180 mL/g. The observed SVI was moderately high which shows the poor settleability of sludge.

The oxygen uptake rate (OUR) was 58.9 mg/L.h. This OUR can be comparable to well working activated sludge process based STPs. The ORP increased from -146 mV in the UASB effluent to 53.5 mV indicating the aerobic nature of the activated sludge process. A summary of treatment performance of the activated sludge post treatment system is presented in Table 3.

### Surface aeration + activated sludge process followed by secondary clarifier

UASB effluent upgrade at 43 MLD, STP at Vadodara was performed by a surface aeration followed by activated sludge process The BOD, COD and SS along with other parameters such as TC and FC were measured in order to determine the treatment performance. The sewage temperature was between 27.5 - 28°C during the monitoring period of the STP. The performance of the surface aeration followed by activated sludge process in terms of BOD and SS removal is presented in Table 3.

### BOD, COD and TSS removals

**Figure 4:**
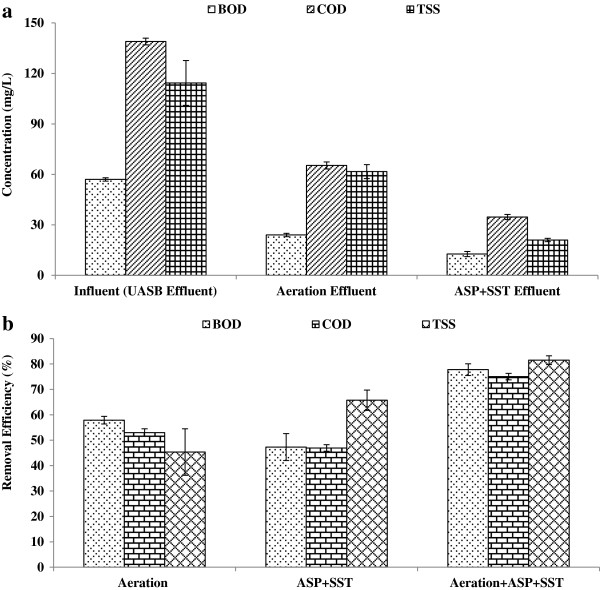
Organics & suspended solids profile of Aeration + ASP system (a) BOD, COD & TSS conc. (b) System performance.

The removal of UASB effluent organics by the surface aeration activated sludge process was high. The mean values of BOD, COD and TSS concentrations of final effluent were 13, 35 and 21 mg/L respectively. The results of this study revealed that the surface aeration + activated sludge is capable to achieve wastewater disposal standards Figure 4 (a & b).

The surface aeration unit was operated at high DO concentration (5-6 mg/L) and short HRT (15 min). Two surface aerators of 10 HP each were imparting the DO uniformly in aeration tank. The short aeration with high DO concentration achieved BOD and COD removal in the flash aeration unit up to 58 and 53%, respectively. The removal of total sulfides was more than 70%. The sulfates concentration increased from 23 to 60 mg/L after the aeration of the UASB effluent.

The main mechanism of the removal of BOD and total sulfides during aeration was the chemical oxidation and stripping of sulfides. Therefore, to gain sufficient confidence of the removal mechanisms in simple aeration of UASB effluent, further studies are needed.

The ASP unit of this post treatment system was operating at MLSS concentration of 2000 to 2500 mg/L with DO levels less than 2 mg/L. The OUR of the activated sludge was evaluated as 50 mg/L.h. The SV30 and SVI were determined equal to 410 mL/L and 164 mL/g, respectively. The SVI shows the good settling properties of the activated sludge. The removal of organics and suspended solids in the ASP process alone was 52 and 73% respectively. The overall process achieved a removal of 92, 94 and 95% for the BOD, COD and TSS, respectively.

### Pathogens removal

The TC and FC concentration decreased from 4.3 × 10^4^ - 9.3 × 10^5^ MPN/100 mL to 4.3 × 10^3^ MPN/100 mL. The removal of TC and FC during aeration and activated sludge process was of the order of 1-2 logs. The removal of TC and FC by the overall process was about 99%. The final effluent coliform counts was still higher than the permissible limit of 1000 MPN/100 mL and needs further treatment. The TC and FC removal mechanisms in the ASP are associated with the predation of higher organisms.

### Nitrogen and Phosphorous removal

**Figure 5:**
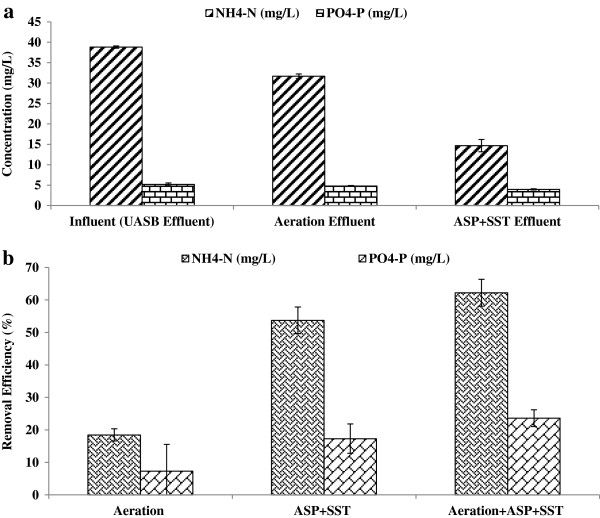
Nitrogen & phosphorous profile of Aeration + ASP system (a) NH4-N & PO4-P conc. (b) System performance.

The final effluent NH_4_-N concentration decreased to 14.7 mg/L implying more than 62% removal Figure 5 (a & b). The final effluent contains still high ammonia nitrogen concerning the disposal to sensitive water bodies. The phosphorus removal was negligible in the system.

#### Aeration + Polishing Ponds

The aeration + polishing pond system is used at three STPs (111, 152 and 48 MLD) at Ludhiana for UASB effluent post treatment. The Ludhiana is a highly industrialized city of India. Due to improper management of sewer system and disposal of untreated industrial wastewater into the sewer, relatively high concentrations of heavy metals were observed. Moreover, the 152 MLD STP also received untreated dairy wastewater which contains high solids concentration.

The mean removal efficiencies for BOD, COD and TSS of the surface aeration + PP system at three STPs are presented in Table 3. The final effluent was characterized by high ammonia nitrogen at all three STPs. Phosphorus removal was also negligible.

### Organics and TSS removals

Results revealed that the performance of the 111 MLD STP was significantly well as compared to the other two STPs viz. 152 and 48 MLD STPs. experience shows that well O & M at this STP enhance the treatment performance. The final effluent BOD concentration decreased to 49 mg/L; COD to 79 mg/L and TSS to 52 mg/L. The surface aeration followed by the PP achieved overall removal efficiency of 14-54, 22-86 and 27-81% for BOD, COD and TSS, respectively. The final effluent concentration was according to the disposal standards. The effluent TSS concentration of the Aeration + PP was 52 ± 18.7, 239 ± 6.0, and 185 ± 55 mg/L at 111, 152 and 48 MLD STPs, respectively. Effluent TSS concentration was high at152 MLD STP and might be due to the sludge wash out. Since the DO in the aeration tank was low (<0.5 mg/L), the stripping and oxidation of sulfides was insignificant. The final sulfides concentration further increased after the polishing ponds which may be attributed to anaerobic conditions and degradation of settleable organic matter.

### Pathogens removal

The removal of TC and FC in FA + PP post treatment system was ~ 1.0 log. The fecal coliform counts in final effluent were 2.3 × 10^3^ to 2.3 × 10^4^ MPN/100 mL, higher than the permissible WHO limit.

## Summary/discussion

The monitoring of 10 STPs of different cities of India was carried out in order to investigate their performance. The primary objective of this study was to assess the treatment performance of full-scale UASB reactors and different post treatment systems. The overall performance of these STPs was ranged from 66 to 95% for BOD, COD and TSS removal (Table 6). However, three UASB reactors at 78, 100 and 48 MLD STPs at Agra, Surat and Ludhiana revealed low treatment efficiency due to poor O & M.

**Table 6:** Summary of treatment performance of all STPs

**STPs location**	**Capacities (MLD)**	**Mean (%) removal efficiencies**		
		**BOD**	**COD**	**TSS**
Saharanpur (UASB + PP)	38	74	76	74
Agra (UASB + PP)	78	75	68	53
Karnal (UASB + PP)	40	74	62	69
Karnal (UASB + DHS)	40	92	93	95
Vadodara (UASB + ASP)	43	92	94	95
Surat (UASB + ASP)	100	92	88	81
Noida (UASB + PP)	27	66	67	71
Noida (UASB + PP)	34	81	71	69
Ludhiana (UASB + Aer + PP)	111	81	75	82
Ludhiana (UASB + Aer + PP)	152	74	75	73
Ludhiana (UASB + Aer + PP)	48	75	70	77

The treatment performance of individual post treatment systems was also evaluated. Results demonstrated that the *DHS and the activated sludge process* were efficient for BOD, COD and TSS removal. The removal of NH_4_-N by DHS and the activated sludge process was significant, but this was not the case for phosphorous. The final effluent BOD and TSS concentrations were in accordance with the disposal standards.

*Polishing ponds*, designed at very low hydraulic retention time (1-2 d) were not capable to remove the organics and nutrients present in UASB effluent. On the other hand, the removal of TSS was high. The nutrients were not affected during post-treatment with polishing ponds. Fecal coliform decrease was also not significant and the final effluent was not in compliance with the disposal standards.

*Aeration followed by PP* was installed at three STPs at Ludhiana. Results of this study revealed that the aeration system at all STPs was not efficient to remove BOD and COD. Low DO concentrations < 1 mg/L and non uniform distribution of dissolved oxygen in aeration tank along with low HRT resulted in poor process performance. The PP in these cases were also designed with low HRT (1 to 2 days), therefore, the overall performance was poor.

The efficiency of STP can be enhanced by incorporating a suitable upcoming post treatment system. There is need to augment the existing STPs, since more stringent standards for biological quality (including nitrogen) cannot be met out by the existing plants. There are a number of post treatment options which are effectively performing well at pilot and demonstration scale reported in literature. Aeration, continuous flow and intermittent decant (CFID) reactor and sequencing batch reactor (SBR) were investigated as post treatment systems of UASB effluent under this research and proposed for further investigation at demonstration scale.

## Conclusions

This short screening study of 10 STPs of seven different cities confirmed that the overall performances of STPs in terms of BOD, COD and SS removal were ranged between 66 to 95%. The performance of UASB reactors at Saharanpur (38 MLD STP), Karnal (40 MLD STP), Vadodara (43 MLD STP) Noida (34 MLD) and Ludhiana (111 MLD STP) were ranged from 55 to 80% for removal of BOD, COD and TSS.

This short investigation also highlighted the performance of different post treatment options presently installed at STP.

• The DHS and the activated sludge process were efficient to remove organics and nitrogen from the UASB effluent. The treated effluent is in conformity to the disposal standards for BOD and TSS.

• Polishing ponds and Aeration + PP systems were performing moderately concerning the removal of BOD and TSS (20-50%). Negligible removal of nutrients was observed in PP and Aeration + PP.

Further, it can be anticipated that there is need to augment the existing STPs, since more stringent standards for effluent quality (including nitrogen) cannot be met.

This short investigation also highlighted the need to conduct screening treatability studies in the event that the sewage stream might contain treatment unknowns that may not be initially apparent. As this investigation was only a qualitative screening study, quantitative kinetic information has not been concluded.

## Competing interests

The authors declare that they have no competing interest.

## Authors’ contributions

All authors contributed significantly to the paper. AAK is main and corresponding author did most of the research work at site and laboratory and performed the statistical analysis. RZG extensively participated in experimental and data analysis, data collection and prepared the initial draft of the manuscript. AA Khan was involved in field studies. IM is the professor who suggested the research program. AAK is another principal investigator who is a professor and guides the research. VD and BL is the faculty member and associated as researcher who coordinated in drafting and designing the manuscript. All authors read and approved the final manuscript.

## Supplementary Material

Additional file 1: Table S1 H_2_S concentration at 111 MLD STP Ludhiana; **Table S2.** H_2_S concentration at 34 MLD STP Noida; **Table S3.** Summary of TSS, VSS & SMA of sludge & biogas production rate at UASB; **Table S4.** Summary of Heavy Metal at three STPs; **Table S5.** Heavy Metal Concentration in Dry UASB Digested Sludge of STPs; **Figure S1.** Variation of influent and effluent BOD of UASB reactor at different STPs; **Figure S2.** Variation of influent and effluent COD of UASB reactor at different STPs; **Figure S3.** Variation of TSS of sewage at different STPs; **Figure S4.** Variation of sulfates in sewage at different STPs; **Figure S5.** Schematic layout of different sampling locations in UASB-PP system at 27; **Figure S6.** Schematic layout of different sampling locations in UASB-PP system at 34 MLD STP, Noida. Click here for file
