# Supplementary material for: Performance assessment of different STPs based on UASB followed by aerobic post treatment systems
Source: J Environ Health Sci Eng. 2014 Jan 27;12:43. doi: 10.1186/2052-336X-12-43 (PMC4108128; doi:10.1186/2052-336X-12-43)
Supplement: Additional file 1: Table S1 — H2S concentration at 111 MLD STP Ludhiana; Table S2. H2S concentration at 34 MLD STP Noida; Table S3. Summary of TSS, VSS & SMA of sludge & biogas production rate at UASB; Table S4. Summary of Heavy Metal at three STPs; Table S5. Heavy Metal Concentration in Dry UASB Digested Sludge of STPs; Figure S1. Variation of influent and effluent BOD of UASB reactor at different STPs; Figure S2. Variation of influent and effluent COD of UASB reactor at different STPs; Figure S3. Variation of TSS of sewage at different STPs; Figure S4. Variation of sulfates in sewage at different STPs; Figure S5. Schematic layout of different sampling locations in UASB-PP system at 27; Figure S6. Schematic layout of different sampling locations in UASB-PP system at 34 MLD STP, Noida. [file 2052-336X-12-43-S1.docx]

**ELECTRONIC SUPPLEMENTARY MATERIAL (TABLES)**

**Table S1 H_2_S concentration at 111 MLD STP Ludhiana**

| **Unit** | **Concentration at inlet (ppm)** | **Concentration at middle (ppm)** | **Concentration at outlet (ppm)** |
| --- | --- | --- | --- |
| Inlet chamber | N.D | N.D | N.D |
| Screens | 0.8 | - | 7.8 |
| Grit chamber | - | - | 0.8/ and 1.6 |
| UASB Reactor inlet | 0.4- 0.6 | 0.4 -0.6 | 1.8- 52.2 |
| Aeration | 1.0-14.0 | 11.4 | 11.4- 14.0 |
| Gas holder | 27.2 | - | - |
| Polishing ponds | 1-14 | 4-5 | 1-5 |

*N.D. - not detected*

**Table S2 H_2_S concentration at 34 MLD STP Noida**

| **Unit** | **Concentration at inlet (ppm)** | **Concentration at middle (ppm)** | **Concentration at outlet (ppm)** |
| --- | --- | --- | --- |
| Inlet chamber | 0.5-1.1 | 0.3-0.8 | 0.2-0.8 |
| Screens | 0.8 | - | 2-4 |
| Grit chamber | 0.3-0.4 | 0.2-0.5 | 0.6-0.9 |
| UASB Reactor inlet | 0.2- 0.9 | 0.6 -1.6 | 9.3- 32.1 |
| Gas holder | 15.5-29 | - | - |
| Polishing ponds | 19-31 | 2-5 | 2-5 |

*N.D. - not detected*

**Table S3 Summary of TSS, VSS & SMA of sludge & biogas production rate at UASB**

| **STP Location** | **Capacities (MLD)** | **OLR (kg BOD/m^3^.d)** | **TSS (g/L)** | **VSS (g/L)** | **SMA (mL CH4/g VSS.d)** | **Biogas generation rate (m^3^/h)** |
| --- | --- | --- | --- | --- | --- | --- |
| Saharanpur | 38 | 0.7-0.8 | 68.8-73.59 | 31.45-34.9 | 154-235 | 90-130 |
| Agra | 78 | - | 69.77 | 34.88 | - | - |
| Karnal | 40 | 0.7-0.9 | - | - | - | 130-197 |
| Vadodara | 43 | 0.6-0.8 | 63.56 | 33.55 | 289 | 150-235 |
| Surat | 100 | - | 45.4 | 29.3 | 194 | - |
| Noida | 27 | - | 41.89 | 34.69 | - | - |
| Noida | 34 | - | 52.34 | 28.7 | - | - |
| Ludhiana | 111 | 0.8-0.9 | 51.81-64.81 | 30.63-33.63 | 270 | 280-380 |
| Ludhiana | 152 | 0.8-1.0 | 55.4-62.5 | 24.5-33.2 | 143 | 129-267 |
| Ludhiana | 48 | 0.7-0.8 | 18.65-116.18 | 9.83-41.89 | 65.6 | - |

*(-) Not detected due to some missing data/ information or malfunctioning of instrument*

**Table S4 Summary of Heavy Metal at three STPs**

| **Heavy Metals** | **111 MLD** | | | **152 MLD** | | | **48 MLD** | | | **Recommended maximum concentration of heavy metals in irrigation waters (mg/L), Metcalf & Eddy, 2005** | **Indian Standard for sewage discharge in inland surface waters (mg/L), CPCB, 2005** |
| --- | --- | --- | --- | --- | --- | --- | --- | --- | --- | --- | --- |
|  | **Sewage** | **UASB Effluent** | **Final Effluent** | **Sewage** | **UASB Effluent** | **Final Effluent** | **Sewage** | **UASB Effluent** | **Final Effluent** |  |  |
| Fe | 3.45 | 1.51 | 0.66 | 9.73 | 0.30 | 1.69 | 9.64 | 1.11 | 0.28 | 5 | 3 |
| Cu | 2.74 | 0.475 | 0.885 | 1.15 | 0.18 | 0.01 | 0.37 | 0.02 | 0.14 | 0.2 | 3 |
| Mn | 7.6 | 0.183 | 0.11 | 0.89 | 0.24 | 0.23 | 0.71 | 0.38 | 0.19 | 0.2 | 2 |
| Co | 0.835 | Nil | 0.385 | 0.01 | 0.00 | Nil | 1.09 | 0.05 | 0.02 | 0.05 | - |
| Pb | 2.55 | 2.16 | 0.76 | 0.06 | 0.06 | 0.04 | 1.76 | 0.08 | 0.05 | 5 | 0.1 |
| Cr | N.D | N.D. | N.D. | 0.56 | 0.16 | N.D. | 1.56 | 0.74 | 0.71 | 0.1 | 2 |
| Zn | 13.4 | 5.22 | 0.79 | 32.7 | 20.01 | 17.30 | 8.06 | 0.12 | 1.53 | 2 | 5 |
| Cd | 0.95 | 0.2 | 0.065 | 0.00 | 0.00 | 0.00 | 0.01 | 0.01 | 0.01 | 0.01 | 2 |
| Ni | 0.865 | 0.73 | 0.63 | 0.26 | 0.30 | 0.01 | 1.31 | 0.08 | 0.41 | 0.2 | 3 |

**Table S5 Heavy Metal Concentration in Dry UASB Digested Sludge of STPs**

| **Parameters** | **unit** | **111 MLD** | | **152 MLD** | | **48 MLD** | | **Concentration of nutrients in anaerobic sewage sludge (EPA, 1983) Polprasert, 1989** | |
| --- | --- | --- | --- | --- | --- | --- | --- | --- | --- |
|  |  | **Concentration** | | **Concentration** | | **Concentration** | | **Range** | **Mean** |
|  |  |  |  |  |  |  |  | **Maximum Permissible Concentration (gm/kg), US EPA 1995 Part 503 Rules (Metcalf & Eddy, 2003)** | **Maximum Permissible Concentration (gm/kg)**  **US EPA, 1983 guidelines for sludge application for fruit & Vegetable application** |
|  |  | **Min.** | **Max.** | **Min.** | **Max.** | **Min.** | **Max.** |  |  |
| Co | g/Kg | Nil | 0.02 | 0 | 0.04 | 0 | 1.56 | - | - |
| Cr | g/Kg | N.D. | 0.69 | 0.41 | 1.04 | 1.1 | 1.867 | - | - |
| Ni | g/Kg | 0.08 | 1.14 | 0.92 | 0.93 | 1.25 | 2.34 | 0.42 | - |
| Cu | g/Kg | 0.24 | 13.5 | 0.34 | 0.49 | 0.21 | 27.3 | 4.3 | - |
| Fe | g/Kg | N.A. | 6.9 | N.D. | 0.17 | 14.8 | 15.2 | - | - |
| Zn | g/Kg | 0.14 | 46.2 | 0.07 | 1.05 | 0.56 | 71.6 | 7.5 | - |
| Mn | g/Kg | 0.18 | 8.7 | 0.19 | 0.20 | 0.28 | 17.31 | - | - |
| Pb | g/Kg | 0.5 | 27.4 | 0.08 | 0.09 | 0.17 | 19.6 | 0.84 | 1 |
| Cd | g/Kg | 0.03 | 1.89 | 0 | 0.01 | 0.12 | 0.23 | 0.09 | 0.025 |

**Electronic supplementary material (figures)**

**Fig. S1 Variation of influent and effluent BOD of UASB reactor at different STPs**

**Fig. S2 Variation of influent and effluent COD of UASB reactor at different STPs**

**Fig. S3 Variation of TSS of sewage at different STPs**

**Fig. S4 Variation of sulfates in sewage at different STPs**

**Fig. S5 Schematic layout of different sampling locations in UASB-PP system at 27 MLD STP, Noida**

**Fig. S6 Schematic layout of different sampling locations in UASB-PP system at 34 MLD STP, Noida**
